# Vis Conservatrix

**Published:** 1867-10

**Authors:** F. R. Payne


ARTICLE LXIV.

### VIS CONSERVATRIX.

BY F. R. PAYNE, M.D.

Read before the Clark County Medical Society, July 1st, 1867.

This is a conservative power, which is alive and opposed to the inroads of all noxious and injurious agents which may be introduced into the body. When medicine was in its infancy, this power was acknowledged by all reflecting men. This knowledge deeply impresses all who have carefully observed it, that "the difference between a good physician and no physician at all, is not half so great as the difference between a good and a bad physician." A few years of careful observation at the bedside of the sick, will induce all true medical men to acknowledge the claims of this celebrated disciple of physic. He was born when organized matter was first created, making his antiquity coeval with that of Adam and Eve. From that day to this, he has been an energetic and self-reliant practitioner.

He has had his triumphs and misfortunes, but never becoming weary in his efforts to prevent the death of all living matter. At an early day, he was the Archæus of Van Helmont, and the Anima of Stahl. His power is surely great, and it is doubtless true that his existence is eternal. This being true, in the days of Van Helmont this power to cure and resist disease was impersonated. He is invisible, but yet lives in all organized matter. He has practiced his profession from the beginning of time, and is not worn out by years, or discouraged by frequent failures. His medicine is always given at the proper time and in proper doses, and is compounded with unparalleled skill, and he commits no errors on account of incorrect diagnosis. All of the various branches of our profession are perfectly understood by this long-bearded and gray-headed Dr. Archæus.

We have innumerable exhibitions of the skill of this great disciple of medicine. When man or beast is exposed to cold, (one of the general causes of disease,) the blood leaves the surface, and the internal organs become engorged, the old Doctor quickly arouses the action of the heart and thus relieves the congestion, and, by so doing, prevents inflammation. If the stomach is loaded with a mixture of incompatible articles of diet, the old physician inverts the action of that organ and discharges the offending matter by vomiting; but if this cannot be done, he gets up a ferment and converts the improper food into a cathartic, and the patient is relieved by its discharge through the bowels. If a malarious or contagious poison is introduced into the system, he carries it off by arousing to increased action some of the secretory organs, and thus prevents its noxious effects. When a grain of sand or dirt enters the eye, how promptly he comes with a flood of tears to wash away the offending substance. When a piece of muscle is removed, by accident or otherwise, how quickly he sends his compound remedies and, with perfect scientific accuracy, replaces the constituent elements which are absent, and thus restores symmetry to the part. If a thorn or some other foreign element enters the flesh, he, not being skilled in the use of surgical instruments, resorts to a more complicated, but none the less effectual, mode of relief; by inflammation and suppuration the extraneous matter is removed.

This antediluvian doctor was truly great, and during the early ages of man he had no compeers. He graduated on the morning of the creation of the world, and has ever been, and will ever continue to be, a practitioner of medicine. Unlike the ordinary members of the profession, the accumulated knowledge of six thousand years has not increased his ability to cure diseases. Many of his patients died, the treatment was not infallible; hence, the necessity for assistants was clearly made manifest.

When the remedy he used for the removal of a grain of sand from the eye fails, the assistant-doctor comes in with his instruments, and by force removes the offending substance. If the stimulus of the blood (whereby cold engorges the heart and internal organs) is not sufficient to relieve the congestion, and thus prevent inflammation, the assistant-doctor renders efficient aid by the use of artificial stimulants. When the system is filled with endemic, epidemic, or contagious poisons, this venerable doctor arouses to increased action the secreting and eliminating powers of nature and thus often throws off the poison and prevents disease. If this treatment is not successful, the assistant-doctor resorts to antiseptics and neutralizing remedial agents, and stops the destructive effects of the poison.

This power, when personified and denominated Dr. Archæus, is skilled in the profession; he never commits an error in diagnosis or treatment; he never boasts of the superior power of his remedies, but moves on quietly in the discharge of his professional duties; he silently and effectually (in many cases) prevents death by the improper use of remedial agents which his assistants employ.

This all being true, he is, unfortunately and necessarily, the main prop and backbone of all false theories and medical imposters. Were it not for his aid, every false theory and medical quack would sink into the most loathsome contempt, not only among the learned but the unlearned. Magic incantation, hocus-pocus, snap doctors, and infallible cancer curers would no longer impose upon the people and disgrace our noble profession; the infinitessimal doses of the homœopath would no longer delude and swindle mankind. Let the silent workings of Dr. Archæus be fully understood, and these delusions would instantaneously be exhonerated from the brain of humanity. When men are threatened with or contract disease, it is far better to leave the case with Dr. Archæus, nature's physician, than to take an improper remedy or preventive.

When the steam-doctor looms out with his heating engine and concentrated decoction of red pepper and smart grass, and makes the blood almost boil in the veins and arteries of deluded mortality, Dr. Archæus silently and quietly steps in with a copious perspiration, and thus puts out the fire and prevents the unfortunate patient from being scalded to death.

The root-doctor rides over the country, boasting of his power over disease. With huge pill-bags and cartloads of noxious vegetable plants, he embarks on his pretended mission of love.

The stomachs of his patients are filled to repletion with infusions, decoctions, and syrups, amply sufficient in themselves to produce disease, were it not for the protecting power of our antediluvian doctor. Some may really be sick, but in a few days get well, not on account of the remedies, but in spite of them. In such cases, Dr. Archæus, who cured the disease and prevented the roots and herbs from killing the patient, gets no praise, but everybody in the neighborhood is ready to bow down and worship the imposter for his matchless professional skill.

Gentlemen of the Society, we have endeavored to briefly point out, in these remarks, the absolute necessity of a thorough knowledge of the conservative power of nature in the prevention and cure of disease. If this is not accomplished, no man ss justified, or can safely administer remedies; he may have a quackish routine course of treatment in all diseases, but will often administer medicine which is incompatible with the remedial agents of nature, and thus destroy the life of his patient. This, all men of extended medical knowledge will admit; consequently, we can clearly see the force of the remark, that the difference a good doctor and no doctor at all, is not so great as the difference between a good and bad doctor. When we are sick, it is much better to take no medicine unless we get that which will aid nature in curing disease. Our country is flooded with patent and secret remedies, and millions of dollars' worth are sold every year, nearly all of which are worse than useless. They not only deplete the bodies of those who take them, but engender disease and impoverish, pecuniarily, the purchasers, and, at the same time, fill the coffers of the dishonest vendor. During the last few weeks, I examined a young man who had, in the last three months, taken twenty-seven dollars worth of patent ague syrups and pills. When he came to my office, the disease was not arrested, but the effects of the remedies he had been using were worse than the ague.

There are thousands of such instances all over the country. The only safe rule is, unless we know what the remedy is and are positively sure that it will not conflict with the curative power of nature, never to let it enter the stomach. If we cannot get the proper medicine, and secure the services of some physician who can give it at the right time and in proper doses, take nothing, and in all probability Dr. Archæus will effect a cure; at any rate, if he fails, we will have the consolation of knowing that the patient was not killed with medicines.

All true friends of humanity, and our profession, hail with gratification the recent action of the American Medical Association, in regard to elevating the standard of medical education. By a unanimous vote of that convention, the minimum duration of college instruction shall be six calendar months, and embrace all of the branches of our science, which shall be divided into three classes or series; and no person can graduate until he has attended three courses of lectures. The classes will be, 1st. Freshman. 2d. Junior. 3d. Senior; and before matriculating for either series, the student will be required to give satisfactory evidence of a good preliminary education. At the close of each term he will be examined upon the branches he has studied, and, if found qualified, will receive a certificate to that effect. At his final examination, these certificates will stand in lieu of an examination on the Freshman and Junior series. The plan of instruction will be similar, in fact nearly the same as that pursued by all of the literary colleges in the country. When this plan for the government of our medical colleges is universally adopted, the quacks who now flood our country will be compelled either to quit the profession or apply themselves to the study of its various branches.

It is hoped that the medical men of America will urge the adoption of this new plan of instruction, and we will then know that students who pass through college will, at least, understand the *vis conservatrix*, and thus be enabled, at all times and under all circumstances, to make rational prescriptions.

The profession is under great obligations to Prof. N. S. DAVIS, for his indefatigable energy in inaugurating and practically testing this new plan of medical instruction. For the last eight years, the curriculum of the CHICAGO MEDICAL COLLEGE has embraced this new method, with long terms, thirteen professorships, and successive order of study.

These remarks, though short and disconnected, are respectfully submitted, with the hope that we will all in future study well the character and system of practice adopted by Dr. Archæus, and become his faithful assistants in the cure and prevention of disease.

